# Supplementary material for: Outpatient Antibiotic Resistance Patterns of Escherichia coli Urinary Isolates Differ by Specialty Type
Source: Microbiol Spectr. 2022 Jun 21;10(4):e02373-21. doi: 10.1128/spectrum.02373-21 (PMC9431218; doi:10.1128/spectrum.02373-21)
Supplement: Supplemental file 1 — Supplemental material. Download spectrum.02373-21-s0001.pdf, PDF file, 0.03 MB [file spectrum.02373-21-s0001.pdf]

Supplementary material

Supplement Table 1. Percent of urinary *Escherichia coli* resistance (%) among specialties; all sexes and all years

Facility specialty	Ampicillin	Amoxicillin-clavulanate	Ceftriaxone	Ciprofloxacin	Gentamicin	Nitrofurantoin	Trimethoprim-sulfa	Imipenem
General family practice	37.1 (6352/17,139)	11.8 (1976/16,810)	2.8 (481/17129)	10.2 (1758/17,252)	5.1 (888/17,252)	2.7 (471/17,239)	18.6 (3197/17,226)	0 (8/17,229)
Internal medicine	34.9 (676/1939)	11.6 (216/1866)	2.7 (53/1935)	12.9 (252/1960)	3.7 (72/1960)	2.5 (49/1960)	16.5 (323/1954)	0.1 (1/1955)
Pediatrics	37.4 (326/872)	9.8 (84/855)	0.8 (7/871)	5.8 (51/878)	3.4 (30/878)	2.1 (18/877)	16.2 (142/878)	0 (0/878)
Obstetrics and gynecology	34.7 (729/2098)	10.7 (217/2032)	1.8 (37/2096)	8.1 (172/2114)	4.0 (85/2114)	3.7 (78/2114)	16.1 (341/2113)	0 (0/2114)
Urology	43.0 (151/351)	16.2 (49/302)	3.7 (13/347)	24.6 (88/357)	9.0 (32/357)	4.8 (17/357)	25.0 (89/356)	0.6 (2/356)
Oncology	37.1 (82/221)	15.9 (33/208)	4.5 (10/220)	18.3 (41/224)	3.6 (8/224)	5.9 (13/222)	15.8 (35/221)	0 (0/221)
All other specialties	36.8 (520/1414)	12.6 (177/1404)	3.8 (53/1412)	13.7 (196/1430)	5.7 (82/1430)	3.1 (45/1429)	19.3 (275/1428)	0 (0/1429)
Total	36.8 (8836/24,034)	11.7 (2752/23,477)	2.7 (654/24,010)	10.6 (2558/24,215)	4.9 (1197/24,215)	2.9 (691/24,198)	18.2 (4402/24,176)	0.05 (11/24,182)
